# Local intra-uterine Ang-(1–7) infusion attenuates PGE_2_ and 6-keto PGF_1α_ in decidualized uterus of pseudopregnant rats

**DOI:** 10.1186/s12958-016-0202-9

**Published:** 2016-10-18

**Authors:** K. Bridget Brosnihan, Victor M. Pulgar, Manish S. Bharadwaj, Liomar A. A. Neves, Liliya M. Yamaleyeva

**Affiliations:** 1 Hypertension and Vascular Research, Wake Forest University School of Medicine, Medical Center Boulevard, Winston Salem, NC 27157-1032 USA; 2 Department of Obstetrics & Gynecology, Wake Forest University School of Medicine, Medical Center Boulevard, Winston Salem, NC 27157-1032 USA; 3 Department of Internal Medicine, Wake Forest School of Medicine, Winston-Salem, NC USA; 4 Biomedical Research Infrastructure Center, Winston Salem State University, Winston-Salem, NC USA

**Keywords:** Prostanoids, Early pregnancy, Angiotensin-(1-7), Decidualization, Apoptosis, Pseudopregnancy

## Abstract

**Background:**

Cyclooxygenase (COX)-derived prostanoids (PGE_2,_ PGI_2_) are important contributors to the process of decidualization. Previous studies showed the presence of Ang-(1-7) in the primary and secondary decidualized zones of the implantation site at early pregnancy. Decreased concentrations of Ang-(1-7) were found in the decidualized uterus compared to the non-decidualized uterus of pseudopregnant rats, suggesting that low levels of Ang-(1-7) are required for successful decidualization at early pregnancy.

**Methods:**

To understand the role of Ang-(1-7) in prostaglandin production in a decidualized uterus, induced by a bolus injection of sesame oil, Ang-(1-7) (24 μg/kg/h) or vehicle was then infused directly into the decidualized uterine horn using an osmotic minipump. The right horns were not injected or infused and served as non-decidualized uterine horns in both groups of animals.

**Results:**

Decidualization increased PGE_2_ concentration in the uterus (0.53 ± 0.05 vs. 12.0 ± 3.2 pmol/mg protein, *p* < 0.001, non-decidualized vs. decidualized horns); Ang-(1-7) infusion attenuated the increase of PGE_2_ (12.0 ± 3.2 vs. 5.1 ± 1.3 pmol/mg protein, *p* < 0.01 control vs. Ang-(1-7) treated decidualized horns). The stable metabolite of PGI_2_ (6-keto PGF_1α_) was increased with decidualization (0.79 ± 0.17 vs. 3.5 ± 0.82 pmol/mg protein, *p* < 0.001, non-decidualized vs. decidualized horns). Ang-(1-7) infusion attenuated the increase in 6-keto PGF_1α_ in the decidualized horn (3.5 ± 0.82 vs 1.8 ± 0.37 pmol/mg protein, *p* < 0.05 control vs. Ang-(1-7) treated decidualized horns). The circulating levels of 6-keto-PGF_1a_ and TXB_2_ were decreased by Ang-(1-7) infusion, while no difference was observed in circulating PGE_2_. Although the global assessment of cleaved caspase 3 immunostaining, a marker of apoptosis, was unchanged within the Ang-(1-7) decidualized horn, there were localized decreases in cleaved caspase 3 staining in the luminal region in the decidualized uterus of Ang-(1-7)-treated rats.

**Conclusions:**

These studies show that increased local uterine Ang-(1-7) alters the uterine prostaglandin environment, possibly leading to disruptions of early events of decidualization.

## Background

The outcome of pregnancy depends on the success of implantation and placentation [1–3]. Cyclooxygenase (COX)-derived prostanoids (PGE_2,_ PGI_2_) possess vasoactive, mitogenic and differentiating properties which regulate numerous maternal-fetal interactions during normal early pregnancy [2, 4]. Gene targeting experiments have demonstrated that COX_2_ derived prostanoids are essential for implantation and decidualization [1, 2, 5]. Prostanoids participate in the regulation of the angiogenic processes, vascular permeability, apoptosis, and remodeling of the endometrium during implantation and decidualization [6–9].

During late pregnancy there is an overexpression of the local placental renin-angiotensin-system (RAS) [10]; however the physiological role of the stimulated RAS is unknown in normal pregnancy and in pathophysiological disorders of pregnancy. Our group provided evidence that the RAS may play an important role during normal pregnancy [10, 11], and an adequate balance of the two main biological active peptides, angiotensin II (Ang II) and angiotensin-(1-7) (Ang-(1-7)) may be essential for the maintenance of normal pregnancy. During early pregnancy Ang-(1-7) staining was found in the implantation site and fetal membranes [11]. Decreased Ang-(1-7) concentration was found in uterine tissue from the implantation site as compared to the interimplantation site at day 7 in pregnant rats [11]. Decreased concentrations of Ang-(1-7) were found in the decidualized uterus compared to the non-decidualized uterus of pseudopregnant rats [12], suggesting that low levels of Ang-(1-7) are required for successful decidualization at early pregnancy. Our overall hypothesis is that Ang-(1-7) exerts an important regulatory role on prostanoid production in the decidualization process of early gestation. In order to test this hypothesis, Ang-(1-7) was infused locally into a decidualized uterine horn of a pseudopregnant rat and its effects on prostanoid concentrations were evaluated.

## Methods

The induction of decidualization and the infusion of Ang-(1-7) into the uterine horn were described in our previously published work focused on the analysis of the effects of Ang-(1-7) treatment on the endocannabinoid system [13]. During these experiments an additional set of uterine tissue was frozen at −80 °C for the analysis of the prostanoids measured in this study. Female Sprague–Dawley rats were obtained from Harlan Laboratories at 10 weeks of age and were ovariectomized under 2 % isofluorane anesthesia. Five days after surgery animals were treated with a hormone regime [17-β estradiol (0.1, 0.2, or 0.3 μg) and progesterone (1 or 4 mg)]; the sequence of hormone delivery is as previously published [13]. On day 5 of hormone treatment animals were anesthetized with 2 % isofluorane and a left horn was injected with a bolus of sesame oil (0.1 ml); an osmotic pump (model 2ML2, pumping rate of 5 μL/h) was implanted subcutaneously on the back and was connected to the left uterine horn for delivery of 24 μg/kg/h of Ang-(1-7) or vehicle of sterile sodium phosphate buffer saline (PBS, pH 7.4) in the two groups of animals. PE60 tubing attached to the minipump was inserted into the uterus lumen until it reached a plastic cuff placed 1 mm from the tip of the catheter. Sutures secured the catheter (before and after the cuff). The right horn was not injected or infused and served as a non-decidualized control in both groups of animals. Five days after surgery and treatment, animals were sacrificed by decapitation. This time point was selected based on studies demonstrating peak uterine mass gain in the pseudopregnant animal [14]. Trunk blood was collected in the presence of indomethacin (20 μg/mL) in the treated and untreated animals. The non-infused and infused uterine horns were removed, weighed, and snap-frozen on dry ice for tissue prostanoid analysis by ELISA. All procedures were approved by the Wake Forest School of Medicine Animal Care and Use Committee.

### Tissue and plasma prostanoids

Frozen tissues were rapidly weighed and homogenized at 20,000 g in a sodium phosphate buffer saline containing inhibitors, EDTA and indomethacin. 2 mL (1 mL sample) and H^3^-PGE_2_ was added for recovery. A 50 μL aliquot of the homogenate was removed for protein determination. 10 mL of acetone was added to 5 ml of homogenate and vortexed. Samples were then centrifuged at 1500 g for 10 min. Acetone was removed by vacuum centrifugation and 3 mL of supernatant were transferred into a 12 × 57 borosilicate glass tube, and dried down to 500 μL (Savant-no heat). This was repeated until 500 μL of solution was left. The remaining samples were acidified to pH 4.0 by adding 50 mM of acetate buffer. The homogenates were extracted and purified using Sep-Pak columns. Columns were activated by rinsing with the 5 mL methanol and 5 mL ultrapure H_2_O. The extracts were assayed using ELISAs for prostaglandin E_2_, 6 keto PGF_1α_ (the stable metabolite of PGI_2_), and TxB_2_ (the stable metabolite of TxA_2_) (Assay Designs, Ann Arbor, MI). Tissue sample content was corrected for recovery and expressed per mg protein. Plasma was extracted (ethanol and acidified with 1 M acetate buffer to pH 4.0), purified using Sep-Pak columns, Sep Pak columns were washed with methanol. After application of the extracted sample to the column, it was washed with ultra-pure water and hexane; then the sample was eluted with ethyl acetate and methanol, evaporated, reconstituted and assayed for PGE_2_, 6-keto PGF1α and TxB_2_ by ELISA (Caymen Chemical, Ann Arbor, MI).

### Immunohistochemistry for Cleaved Caspase-3

The uterus was fixed in 4 % paraformaldehyde, embedded and slides were stained using alkaline phosphatase detection with an antibody to a marker of apoptosis cleaved caspase-3 at a 1:50 dilution (Cell Signaling, Denvesa, MA). Six slides from each group were analyzed. Semi-quantitative analysis was conducted based on grading the intensity of staining from zero (no staining) to four (high intensity) staining. The scoring was performed blinded to the treatment. One hundred cells/slide from four areas (lumen-antimesometrial, lumen-mesometrial, decidual-mesometrial, decidual-antimesometrial) were scored using a 1–3 Micro-Imaging Program that connected to a 60× microscope.

Data were analyzed using a two way analysis of variance (2-way ANOVA) followed by Bonferroni’s post hoc test (GraphPad Software, San Diego, CA) or student t test. A *p* value of less than 0.05 was considered statistically different. All data were expressed as mean ± SEM.

## Results

**Fig. 1:**
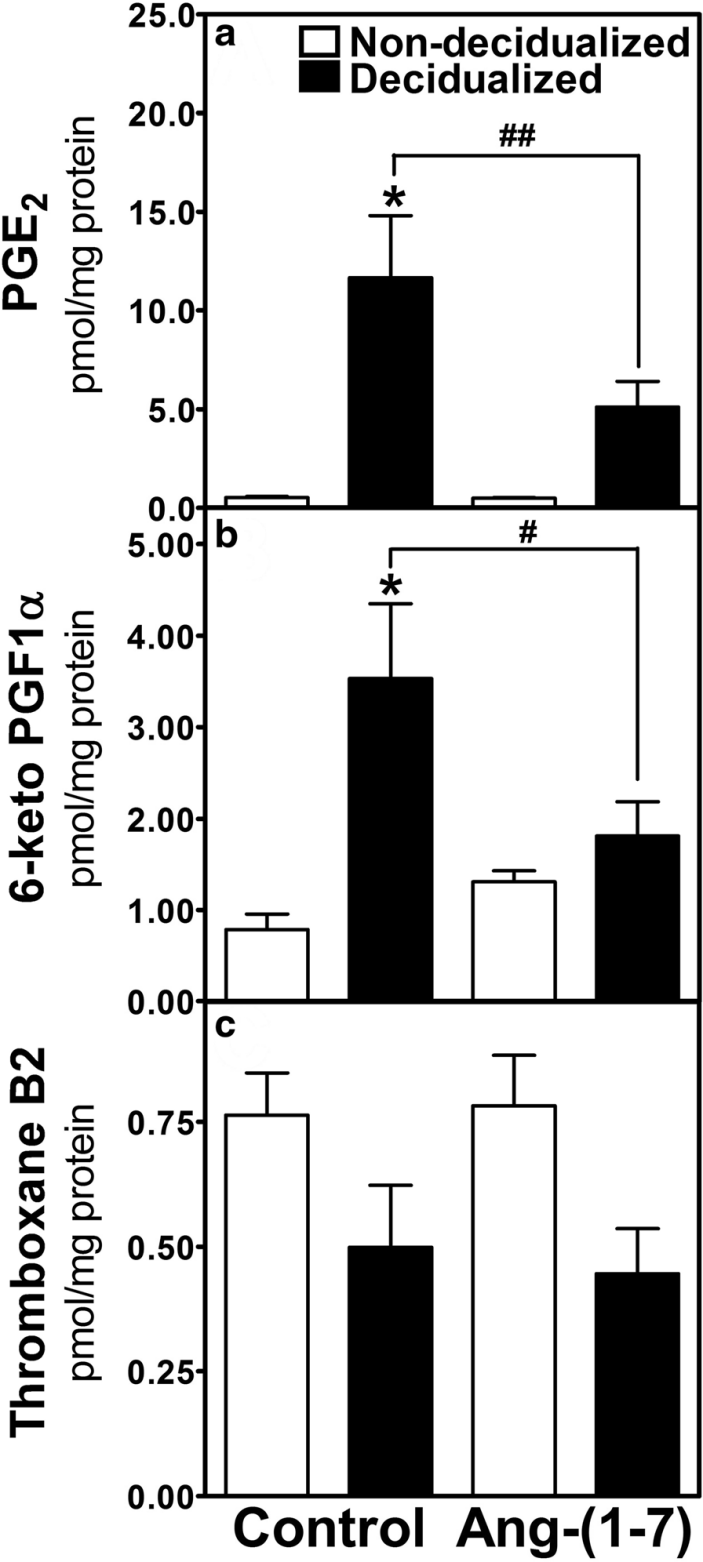
Tissue levels of PGE_2_, (**a**) 6-keto PGF_1α_, (**b**) and TxB_2_ (**c**) (pmol/mg protein) in the non-decidualized and decidualized uterine horns of control and Ang-(1-7) treated animals. The decidualized horn of the two groups of animals received locally infused either control (PBS) or Ang-(1-7) treatment, whereas the non-decidualized horn of each was untreated. Data are mean ± SEM, *n* = 9–10, *p < 0.001 (control non-decidualized vs. decidualized horns) #*p* < 0.05, ##*p* < 0.01 (control decidualized vs. Ang-(1-7) decidualized horns)

In control animals, decidualization was characterized by a 22.2–fold increase in PGE_2_ levels, 4.4-fold increase in 6-keto PGF_1α_, and no significant change in TxB_2_ levels (Fig. 1) as compared to the non-decidualized horn. The predominant prostanoid in the decidualized horn was PGE_2_ > 6-keto PGF_1α_ > TxB_2_, whereas in the non-decidualized uterine horn there was no difference in the content of the three prostanoids, each approximating about 1 pmol/mg protein. Ang-(1-7) treatment (Fig. 1a) into the decidualized horn decreased PGE_2_ levels as compared to the control decidualized horn (*p* < 0.01). Ang-(1-7) treatment also decreased the levels of 6-keto PGF_1α_ as compared to the control decidualized horn (Fig. 1b). There were no significant differences between the non-decidualized and decidualized horns in TxB_2_ levels following Ang-(1-7) treatment (Fig. 1c). There were no differences between the non-decidualized horns in the control and Ang-(1-7) treated groups.

**Fig. 2:**
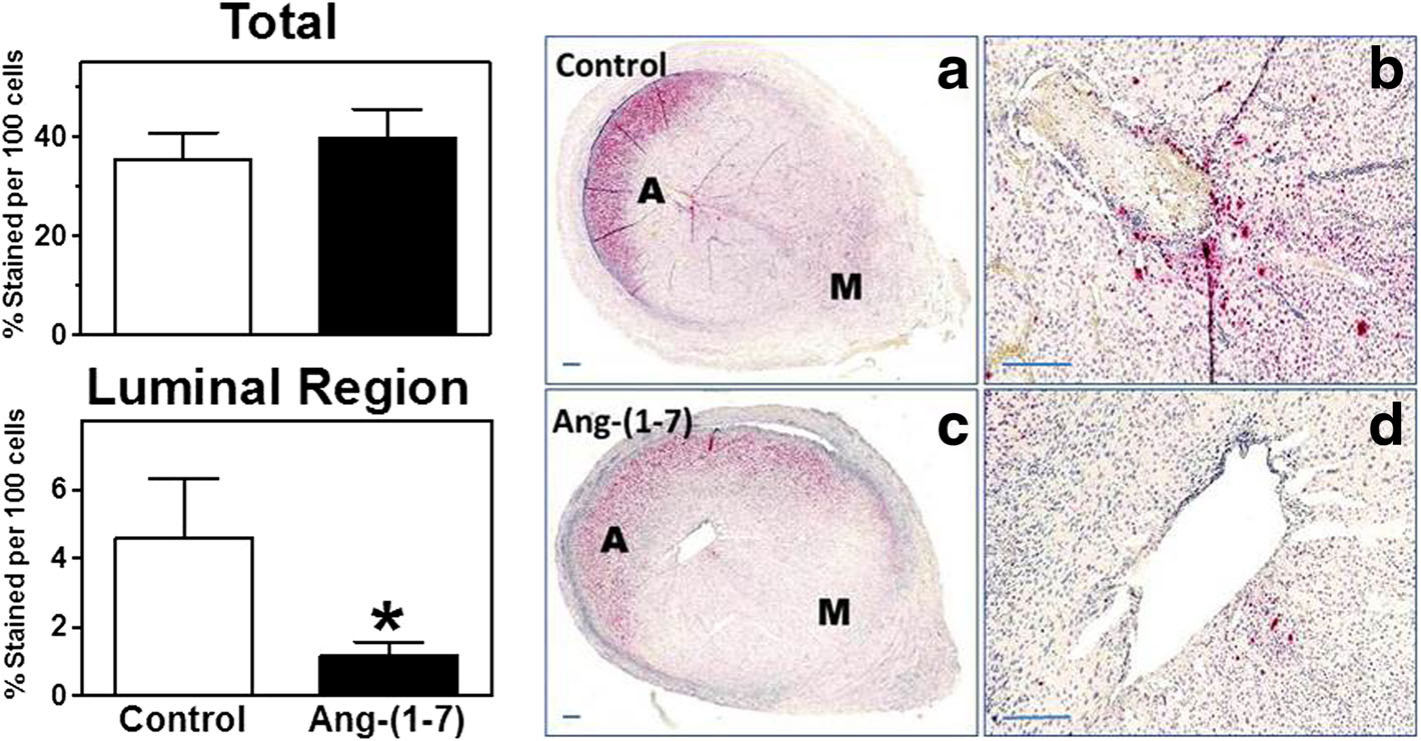
Semi-quantitative assessment of staining of cleaved caspase-3, an apoptotic marker, was determined in total and luminal regions of the control and Ang-(1-7) dedicualized uterus. The % of apoptotic cells when all four regions (luminal and decidual, antimesometrial and mesometrial) (Total) were combined was not different between control and Ang-(1-7)-treated groups, whereas the % of the stained apoptotic cells in the mesometrial and antimesometrial luminal regions (Luminal Region) was reduced with Ang-(1-7) treatment. *n* = 6 animals/group). Representative immunohistochemical cleaved caspase 3 staining of the control (**a** and **b**) and Ang-(1-7) (**c** and **d**) decidualized uterus. A = antimesometrial; M = mesometrial. Scale bar = 200 μm

Our previous study reported no effect of Ang-(1-7) treatment on gene expression of markers of apoptosis, including caspase-3 mRNA [13]. In order to evaluate regional distributional changes in caspase-3 associated with Ang-(1-7) treatment in the uterus, we performed immunostaining for cleaved caspase-3. Our data show no signal for cleaved caspase-3 in the non-decidualized horn. Global assessment of the staining in the four regions of the decidualized horn revealed that Ang-(1-7) treatment had no effect (Fig. 2 Total). The region with the greatest staining was in the antimesometrial decidual region (Fig. 2a and c), but semi-quantative assessment of staining was not changed by Ang-(1-7) (55.2 ± 12.6 vs. 70.7 ± 8.4 % apoptoic cell/100 cells, ns) in this region. However, local assessment of the luminal cells in both the antimesometrial and mesometrial regions showed a significant reduction in apoptosis measured by high intensity cleaved caspase-3 immunostaining with Ang-(1-7) treatment (Fig. 2 Luminal Region and Fig. 2b and d).

**Fig. 3:**
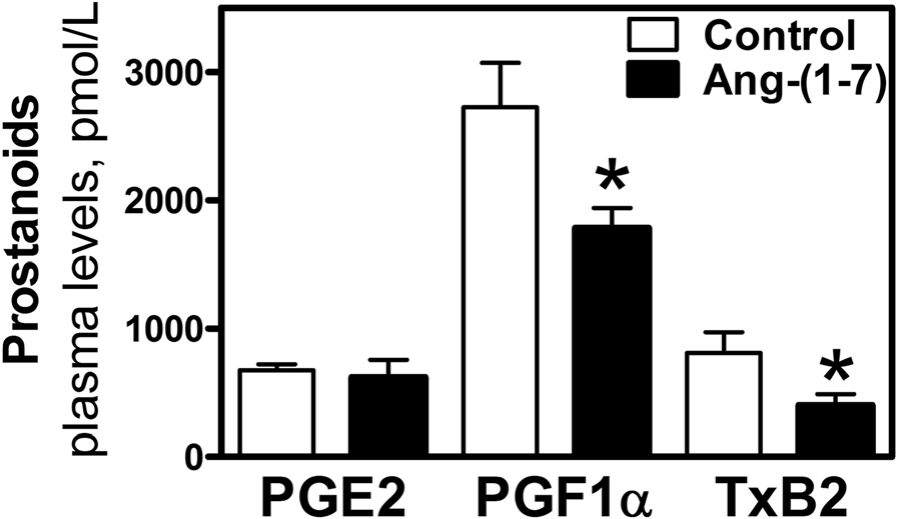
Plasma prostanoids (PGE_2_, 6-keto PGF_1α_, and TxB_2_) in animals that received local intrauterine infused control (PBS) or Ang-(1-7) treatment. Data are mean ± SEM. *n* = 7–10, *p < 0.05 vs. Control

In order to determine if the local uterine infusion of Ang-(1-7) as compared to PBS in the decidualized horns affected circulating levels of prostaglandins, plasma PGE_2_, 6-keto PGF_1α_, and TxB_2_ were compared in the two groups of rats (Fig. 3). The group receiving intra-uterine Ang-(1-7) into the decidualized horn showed reduced plasma 6-keto PGF_1α_ and TxB_2_ levels without a change in PGE_2_ levels (Fig. 3). As previously published [13], circulating levels of Ang I (38 ± 4 vs. 35 ± 6 pmol/L, control vs. Ang-(1-7)) and Ang II (32 ± 3.5 vs. 24 ± 1.5 pmol/L, control vs Ang-(1-7)) were unchanged by the intra-uterine infusion of Ang-(1-7), but there was an increase in circulating levels of Ang-(1-7) (180 ± 28 vs. 320 ± 55 pmol/L, *p* < 0.05 control vs. Ang-(1-7) infused).

## Discussion

Prostanoids are important components of decidualization whereby the maternal uterus is remodeled to become receptive to the blastocyst. Prostaglandins have an obligatory role in the early pregnancy, as evidenced by indomethacin abolishing or significantly delaying implantation and decidualization. Although decidualization usually occurs in conjunction with receiving the implanting blastocyst, mechanical stimulation of the uterine luminal surface in pseudopregnant rodents can induce differentiation of uterine stromal cells into decidual cells in a manner that is similar to blastocyst implantation. In this study we showed that PGE_2_ and 6-keto PGF_1α_ tissue contents are significantly increased in the decidualized uterus of the pseudopregnant rat, without a change in TxB_2_ content. Administration of Ang-(1-7) locally into the decidualized uterus results in significant reduction in the concentration of PGE_2_ and 6-keto PGF_1α_ suggesting that a local increase in Ang-(1-7) may interfere with some aspects of prostaglandin dependent events of decidualization.

In light of our previous work demonstrating that decreased concentrations of Ang II and Ang-(1-7) were found in the decidualized uterus and in the implantation versus inter-implantation sites of early pregnancy and pseudopregnancy [11, 12], our current study shows that exposure to higher levels of local uterine Ang-(1-7) changes the prostanoid profile of the uterus and suggests that an activated RAS alters the normal profile of prostanoids in early pregnancy. The mechanism for this effect may be an action of Ang-(1-7) on the regulation of the synthetic enzymes of the prostanoids, including COX_2_, PGE synthase and/or PGI synthase at the level of the gene or protein. Future studies are required to determine the site of regulation.

As we previously published [13], there was a 9.6 fold increase in weight in the control decidualized horn as compared to the non-decidualized horn (1722 ± 103 vs 179.7 ± 7.1 mg, *p* < 0.001); the weight of the decidualized horn was not altered by Ang-(1-7) infusion (1674 ± 55.9 mg). Permeability measured as the cpm of radiolabeled I^125^ albumin in the uterus relative to the specific activity of skeletal muscle albumin increased with decidualization (7.1 ± 0.5 vs 3.1 ± 0.2 cpm uterus/cpm skeletal muscle, *p* < 0.01), but was unchanged in the decidualized horn with Ang-(1-7) treatment (7.5 ± 0.9 cpm uterus/cpm skeletal muscle) [13]. As previously published, immunostaining of vimentin, a cellular marker of decidualized cells, clearly revealed distinct patterns in the intensity of staining in the decidualized uterus with the mesometrial pole showing more intense staining than the antimesometrial pole; however, there was no effect of Ang-(1-7) on the intensity of staining of vimentin in the decidualized horn [13]. In association with the increase in permeability of the decidualized horn, VEGF mRNA was increased: however, in contrast to the other indices of decidualization, Ang-(1-7) treatment resulted in a further increase in VEGF mRNA in association with the decrease in PGE_2_ and PGF_1α_. The increase in VEGF mRNA is consistent with both its angiogenic and permeability increasing properties [15]. The increase in uterine weight and permeability were accompanied with increases in PGE_1_ and 6-keto PGF_1α_ consistent with previous reports for their involvement in the events of decidualization [9]. The earliest events of pregnancy such as permeability and increase in the uterine mass can be induced by local uterine PGE_2_ infusion [9]. The fact that Ang-(1-7) reduced PGE_2_ and 6-keto PGF_1α_ without a change in weight and permeability was unexpected in light of previous reports by Hamilton et al. [9] who demonstrated that blockade of PGE_1_ and PGF_1α_ using indomethacin reduced decidualized uterine weight and permeability. One explanation for the difference in our and their findings is that Ang-(1-7) only partially reduced PGE_2_ and 6-keo PGF_1α_ and these levels of reduction may not be sufficient to affect an overall change in uterine weight and permeability. Our study does not eliminate the possibility that Ang-(1-7) could be causing other local changes in association with decidualization. For example, measurement of regional changes in a marker of apoptosis revealed localized changes in the decidualized uterus in the luminal region. Ang-(1-7) treatment affects local apoptosis without causing a change in the total uterine weight, permeability, or the total tissue content of mRNA caspase-3. That an increased Ang-(1-7) may be detrimental in early pregnancy is consistent with our observation of its increase in human placenta at the 1^st^ trimester of aborted pregnancy [16]. Further studies are required to assess how the production of prostaglandins was attenuated by increased levels of Ang-(1-7) and whether local changes of Ang-(1-7) and prostanoids in the implantation site are important in influencing the success of implantation. Recent work in rodent models has described a prominent role of prostanoid levels in decidua during the pathogenesis of preeclampsia [17]. In their model of preeclampsia the BPH/5 mice, a single administration of Cox2 inhibitor during early (E6.5 days) decidualization improved fetal growth and attenuated late gestational hypertension. Future studies will be important in determining the balance of RAS and prostanoid levels in adverse pregnancy outcomes, such as preeclampsia.

Circulating levels of PGF_1α_ and TxB_2_ were decreased with Ang-(1-7) local uterine infusion, whereas plasma PGE_2_ did not change. In our previous publication we demonstrated that local uterine infusion of Ang-(1-7) is associated with increased circulating levels of Ang-(1-7) [13]. The reduction in circulating levels of PGF_1α_ is consistent with the reduction in its uterine content with Ang-(1-7) local infusion and could be explained by the reduced release into the circulation rather than a direct effect of the increased circulating Ang-(1-7) with the infusion. Comparison of the biochemical changes in non-decidualized horns in animals with and without Ang-(1-7) treatment in this and our previous study [13] showed that there were comparable levels of PGE_2_, 6-keto- PGF1α, and TxB2, demonstrating that this spill over into the circulation from the infused horn had no effect on the non-decidualized horns. Because TxB_2_ tissue content was not significantly affected by Ang-(1-7), its reduction in the circulation may be due to systemic effects of Ang-(1-7) on TxB_2_. Our data demonstrate that the reduction in uterine PGE_2_ with Ang-(1-7) is not reflected in plasma PGE_2_ levels.

## Conclusions

In summary, these studies demonstrate that elevated Ang-(1-7) in the uterine decidualized horn alters the profile of uterine prostanoids, PGE_2_ and 6-keto PGF_1α_. Local apoptosis in the luminal region was reduced with Ang-(1-7). The direct connection of these events to prostaglandins is yet to be determined. Because of the critical role of prostanoids in the early events of pregnancy, the down-regulation of prostanoids by elevated Ang-(1-7) may lead to complications in the early events of pregnancy.

## Abbreviations

6-keto PGF_1α_: 6-keto prostaglandin PGF_1α_
Ang II: Angiotensin II
Ang-(1-7): Angiotensin-(1-7)
COX: Cyclooxygenase
PGE_2_: Prostaglandin E_2_
RAS: Renin-angiotensin system
TxB_2_: Thromboxane B_2_
