# Erratum to: Local intra-uterine Ang-(1–7) infusion attenuates PGE2 and 6-keto PGF1α in decidualized uterus of pseudopregnant rats

**DOI:** 10.1186/s12958-017-0238-5

**Published:** 2017-03-27

**Authors:** K. Bridget Brosnihan, Victor M. Pulgar, Manish S. Bharadwaj, Liomar A. A. Neves, Liliya M. Yamaleyeva

**Affiliations:** 1 Hypertension and Vascular Research, Wake Forest University School of Medicine, Winston Salem, USA; 2 Department of Obstetrics & Gynecology, Wake Forest University School of Medicine, Winston Salem, USA; 3 Biomedical Research Infrastructure Center, Winston Salem State University, Winston Salem, USA; 4 Department of Internal Medicine, Wake Forest School of Medicine, Winston Salem, USA

## Erratum

Upon publication of the original article [1], on PubMed the author’s name “Victor M. Pulgar” was formatted incorrectly in the XML mark up and therefore appeared incorrectly on PubMed. In this XML mark up, the middle initial “M” was added as a Particle when it should have been included as a Given Name. The author’s name was incorrectly formatted in PubMed due to this error as “M Pulgar V” and not as “Pulgar VM”. The author’s name appears correctly on the BioMed Central website.
